# Immunolocalization of Aquaporin 1, 2, and 9 in Anuran Testis of the Neotropical Pointedbelly Frog *Leptodactylus podicipinus*

**DOI:** 10.3390/cimb46090594

**Published:** 2024-09-10

**Authors:** Rafael O. A. Bordin, Classius de Oliveira, Raquel F. Domeniconi

**Affiliations:** 1 Department of Structural and Functional Biology, Universidade Estadual Paulista “Júlio de Mesquita Filho”, Campus of Botucatu, São Paulo 18618689, SP, Brazil; rafaoliverbordin@gmail.com; 2 Department of Biology, Universidade Estadual Paulista “Júlio de Mesquita Filho”, Campus of São José do Rio Preto, São Paulo 15054000, SP, Brazil; classius.oliveira@unesp.br

**Keywords:** anura, aquaporin, immunohistochemistry, spermatogenesis

## Abstract

Many anuran survival strategies involve hydric regulation, and reproduction is not different. The aquaporin (AQP) family plays an important role in water transport and regulation in many tissues, including the male gonad. The testes undergo various stages of change during the reproductive cycle, and water balance is an important factor for ensuring reproductive success. Considering the relevance of water control in testicular development in anurans and the lack of research regarding the tissue localization of AQP in the male gonad, the present study investigated the expression of three AQPs (1, 2, and 9) in the testis of the neotropical anuran species *Leptodactylus podicipinus* during two different periods of the reproductive cycle (reproductive and non-reproductive). AQP1 and 2 immunoreactions were found in early germ cells, spermatozoa, Leydig cells, and Sertoli cells, which were more frequently expressed within the reproductive period. AQP1 was also found in the testicular blood vessels. AQP9 was identified predominantly in the epithelium of the intratesticular ducts of reproductive-period individuals. This study presents, for the first time, the localization of AQP1, AQP2, and AQP9 in the testes of an anuran species and the differences in their location during two distinct periods of the reproductive cycle.

## 1. Introduction

Water plays a crucial role in the reproductive process of Anura, as these amphibians are highly vulnerable to desiccation due to their skin permeability and reliance on humid environments. In addition, frog eggs are delicate and permeable, making water an essential component for their survival [1]. The natural history of anurans delineates the evolution of diverse reproductive modes, ranging from aquatic to terrestrial, and includes instances of reversion from terrestrial to aquatic habitats [2,3,4]. In the transition of organisms from aquatic to terrestrial environments, the establishment of effective hydric regulation mechanisms is crucial for maintaining optimal water balance. These mechanisms encompass adaptations, including the modulation of egg layer quantity and size, production of foam to prevent dehydration, and preference for reproductive activities in humid locales [5]. Therefore, proper osmotic management is essential for successful reproductive modes [6,7].

The mechanisms responsible for water dependence and regulation are crucial for survival and reproductive success in offspring and female eggs [8,9,10]. However, little information is available regarding the cellular physiology involved in the process of spermatogenesis, which contributes morphologically by producing viable spermatozoa. Understanding the intricate processes of spermatogenesis at the cellular level is essential for gaining comprehensive insight into reproductive biology. This involves not only the production of viable spermatozoa but also the regulation of their development, differentiation, and eventual function during fertilization.

The interaction between physiology and external factors modulates the reproductive cycles of frog species, as reported previously [11,12]. Factors such as temperature, humidity, and seasonal changes significantly influence reproductive patterns and success rates. Moreover, seasonal and local water availability dynamics are fundamental to the development of anuran species [13,14]. These dynamics can affect breeding sites, larval development, and adult survival, highlighting the importance of water as a critical resource. To fully understand the complexity of anuran reproduction, it is necessary to understand the physiology of how anurans regulate and balance osmotic levels in their environment [15]. This includes studying the osmoregulatory mechanisms that allow homeostasis despite fluctuations in external conditions.

Anuran survival is heavily dependent on the process of osmotic regulation, with aquaporins (AQPs) assuming a pivotal role in facilitating osmoregulation. AQPs participate in the management and control of water flux both inside and outside cells, thereby preserving the internal fluid balance of this taxonomic group [15,16,17]. These proteins function via active transport and show different permeabilities to water and other metabolic molecules [18,19]. 

AQPs comprise a family of small “membrane-spanning” proteins with monomers of 26 to 34 kDa. These proteins are expressed in the plasma membranes of cells involved in fluid transport. Proteins belonging to the aquaporin family are homotetramers in the membrane. Each monomer consists of six domains of “membrane-spanning” α-helix with carboxy and aminoterminal extremities oriented to the cytoplasm containing a distinct water pore acting as a water channel that facilitates and regulates the passage through the cell membrane [20]. Aquaporins are present in the membranes of intracellular organelles, regulating the volume of both the organelles and the overall cell [21]. AQPs are involved in many physiological processes such as water and solute homeostasis, secretion, facilitation of transepithelial transport, cellular migration, and neuroexcitation, and they also participate in a variety of pathological processes such as glaucoma, epilepsy, obesity, and cancer [22,23].

In the male genital system, water is necessary for maintaining the luminal environment in which spermatozoa are located. Water functions as a vehicle to transport spermatozoa from the testis to the vas deferens, ensuring that they remain viable and capable of fertilization [24]. The presence and proper functioning of water channels, such as AQPs, are crucial for this process. AQPs facilitate water movement across cell membranes, thereby maintaining the appropriate hydration levels within the reproductive ducts. Different types of aquaporin are expressed in specific regions or cells throughout the extratesticular ducts of mammals. Hermo and Smith [24] reported that AQPs are located in specific membrane domains, including microvilli, apical, basal, and lateral regions of the cell, as well as in endosomes. This strategic localization allows AQPs to efficiently regulate water transport and maintain the osmotic balance required for sperm maturation and mobility. The expression of these AQPs can be controlled by hormonal or luminal factors, indicating a complex regulatory mechanism that ensures optimal reproductive function.

The regulation of AQPs is a multifaceted process that can be influenced by both external and internal factors. AQPs may also be regulated and influenced by external and internal factors such as variations in pH, phosphorylation, and auxiliary proteins [20,25,26,27,28,29,30]. There is evidence of a direct relationship between aquaporin differential expression and environmental water availability [15,31,32]. These regulatory mechanisms are essential for adapting to changing physiological conditions and maintaining reproductive health.

The success of the biological processes of anurans is intimately bound to the hydric dynamics, osmotic regulation, and physiological control of water flux through organs [15,16]. Evidence indicates that the dynamics of fluid concentrations between the internal and external mediums plays a fundamental role in biological processes such as reproduction and in the success of external fertilization, especially regarding the activation of sperm motility in frogs [17,27]. Considering this information, water is important in the dynamics of the male genital system, and water regulation in the testis by aquaporin is an essential process for sperm concentration [33,34] and consequential to male fertility [35,36].

In light of the pivotal role aquaporin play in water regulation in the male reproductive system [19] and the lack of research on the expression of aquaporin across different animal groups and environmental context, particularly in anuran reproduction [15], this study seeks to investigate the expression patterns of three distinct aquaporin proteins (AQP1, 2, and 9) in the testis of the neotropical anuran species *Leptodactylus podicipinus* throughout its reproductive cycle. Therefore, this study aimed to elucidate the localization of these proteins within testicular tissue and determine whether differences exist in the labeling of aquaporins throughout the species’ reproductive cycle.

## 2. Materials and Methods

### 2.1. Sampling and Location

Animals were obtained from specific areas for the reproduction of species [37,38]. Rio Bonito (22°40′28.0″ S 48°20′00.8″ W) and Porto Said (22°40′49.1″ S 48°19′37.0″ W) are both located in the city of Botucatu state of São Paulo, Brazil.

The sampling was carried out through monthly expeditions over two years (2021–2023), totalizing 8 mature males separated into two groups (reproductive, n = 4; non-reproductive, n = 4) representing different periods of the species’ reproductive cycle. The periods were determined according to species vocalization activities and seasonal rainfall at the study site. The presence of secondary sexual characters such as an apparent vocal sac and nuptial thorns in the thoracic limb confirmed sexual maturity. The location and capture of specimens were performed via active searches at vocalization sites during the crepuscular period. The specimens used in the experiment were treated according to the Ethical Principles of Animal Experimentation adopted by the Colégio Brasileiro de Experimentação Animal (CEUA N° 4478221021). All expeditions and sampling were conducted with a sampling license (n° 79953-3 SISBIO/IBAMA).

### 2.2. Immunohistochemistry

After euthanasia with topical application of 5% benzocaine, the gonads were fixed for 24 h by immersion in Tamponade Formalin 10% (90% PBS, 10% Formol) at room temperature. After fixation, the animals were submitted to inclusion in Paraplast (Sigma-Aldrich^®^, St. Louis, MO, USA), and 3 non-consecutive longitudinal sections were made per animal (3 μm thick, 50 μm distance between the sections).

**Figure A1:**
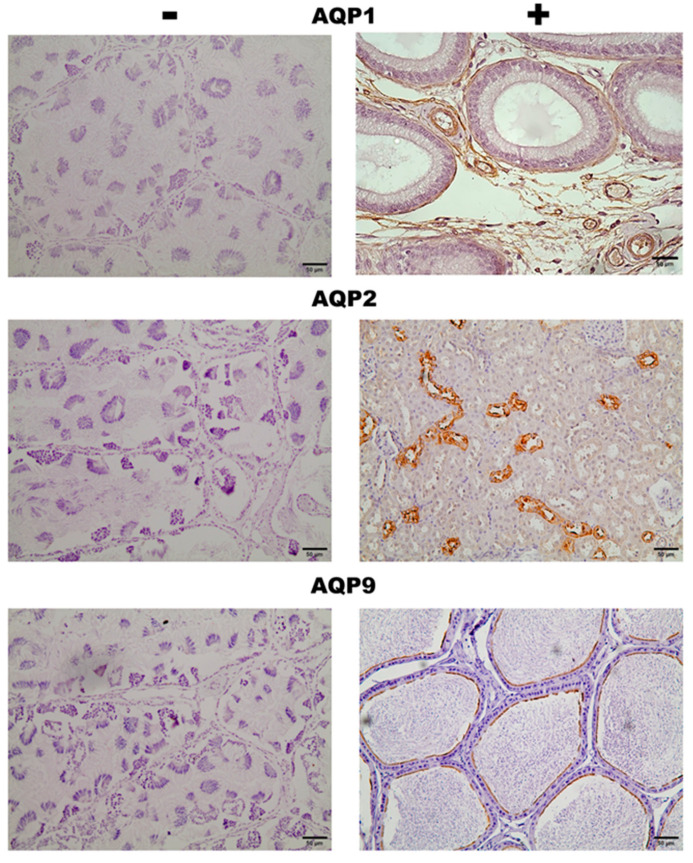
Immunohistochemistry staining of positive and negative controls. Negative controls: AQP1, AQP2, and AQP9 were performed using testis tissue from *Leptodactylus podicipinus*. Positive controls: AQP1: Rat epididymis presenting specific labeling on blood vessels; AQP2: Rat kidney with labeling on the collecting ducts; AQP9: Mouse epididymis with labeling on the tubule stereocilia. Magnification 20× bar = 50 μm.

Histological slides of the testes were prepared, first being deparaffinized in xylol followed by dehydration in ethanol. Antigenic retrieval was performed by subjecting the slides to immersion in tamponade citrate solution (pH 6.0) and subsequent exposure to a temperature of 100 °C for 15 min in a microwave (750 W). To ensure optimal results, peroxidase blockade was performed in methanol containing 3% H_2_O_2_ for 15 min. To reduce the nonspecific link, slides were incubated in a blocking solution comprising bovine serum albumin (BSA) at a concentration of 3% in PBS-T for 1 h at room temperature. Following preparatory steps, the detection of AQP1, AQP2, and AQP9 was meticulously carried out using selected antibody dilutions at a concentration of 1:100 (Anti-AQP1 AB2219 Sigma-Aldrich; Anti-AQP2 AB3066 Sigma-Aldrich; Anti-Aqp9 AQP91-A Alpha Diagnostics, San Antonio, TX, USA) diluted in PBS, with overnight incubation at 4 °C. Subsequent to thorough washing with PBS, the slides were incubated with a secondary HRP antibody (Anti-rabbit IgG—1:100 AB6721 Abcam, Waltham, MA, USA) for 1 h at room temperature, followed by revelation using diaminobenzidine (DAB) and coloration with Mayer Hematoxilyn. Negative controls were generated by substituting the primary antibody for 0.01 M of PBS. Positive controls were employed to validate specific labeling using slides containing mouse and rat tissues (Figure A1, Appendix A).

Commercially available antibodies were used because aquaporin from anuran tissue presents a high homology to mammalian aquaporins (AQP1 60% similarity [16,39]; AQP2 70% similarity [16,40]).

After immunohistochemistry, the slides were coded for blinded analysis. The evaluation of the AQP1, 2, and 9 reaction results outcomes entailed meticulous comparison of reaction intensities across the two designated period groups. This analysis was conducted by Bordin and was independently corroborated by Dr. Domeniconi to ensure rigor and reliability. Subsequently, the coded slides were photographed under a light microscope (Axiophot 2) equipped with a digital camera AxioCam HR (Zeiss, Jena, Germany) from the Department of Structural and Functional Biology, Anatomy sector, Biosciences Institute, UNESP—State University of São Paulo, Brazil.

### 2.3. Western Blotting

Frozen testicles were homogenized (n = 9; 5 reproductive and 4 non-reproductive) using a sonicating homogenizer (Q125 Sonicator Qsonica Inc., Newtown, CT, USA), with RIPA lysis buffer (Bio-Rad, Hercules, CA, USA) enhanced with a protease inhibitor mix (Sigma, St. Louis, MO, USA) for 5 min, with a cycle of 1 min per pulse at 40% amplitude while maintaining the temperature at 4 °C. Following this process, the samples were centrifuged to separate the cellular debris, and the protein fraction was obtained from the resulting supernatant. Protein concentrations in the samples were then determined using the Bradford method. Subsequently, the samples (60 μg per sample), with the addition of Laemmli buffer (1:1 dilution), were heated in a dry bath at 96 °C for 15 min to denature the proteins. These samples were then loaded onto 10% SDS-polyacrylamide gels for electrophoresis under reducing conditions. Post-electrophoresis, the separated protein bands were transferred onto a nitrocellulose membrane (Sigma). The membrane was blocked with 3% non-fat dry milk in TBST (comprising 10 mM TRIS-HCl pH 7.5, 150 mM NaCl, 0.1% Tween-20) for 1 h to prevent non-specific binding. The blot was then incubated overnight at 4 °C with primary antibodies against AQP1 (AB2219, Sigma-Aldrich), AQP2 (AB3066, Sigma-Aldrich), or AQP9 (Aqp91-A, Alpha Diagnostic), each diluted at a ratio of 1:1000 in 3% bovine serum albumin. Following this incubation, the blot membrane was washed with TBST seven times for 5 min to remove unbound antibodies. The samples were then incubated for 1 h at room temperature with peroxidase-conjugated goat anti-rabbit-IgG secondary antibody (AB6721, Abcam). After a subsequent series of seven 5 min washes in TBST, the proteins were visualized using an ECL prime detection reagent (Sigma). Beta-actin was used as a positive control for quality control and to ensure equal loading (Figure A1; Appendix A).

### 2.4. Statistical Analysis

The blot images were analyzed and quantified using ImageJ v1.54. The mean of each lane was compared between periods using Student’s *t*-test or Mann–Whitney test, based on data distribution, with a significance level of *p* < 0.05. All statistical analyses were performed using GraphPad Prism 8 software.

## 3. Results

**Figure 1:**
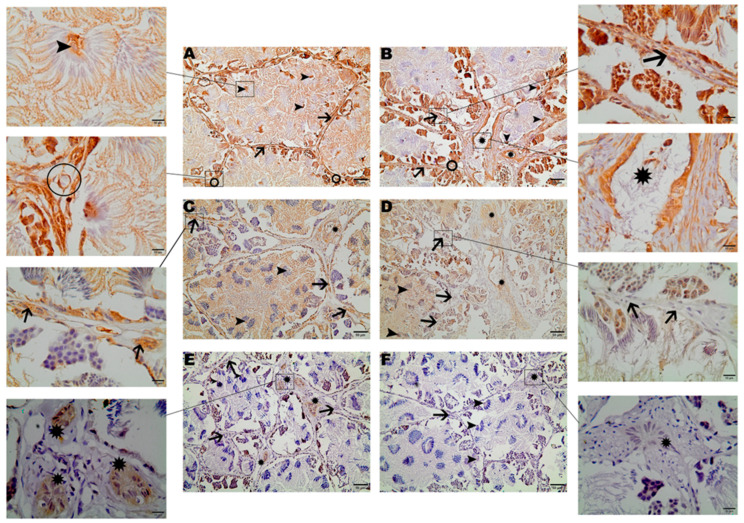
Immunohistochemical staining of AQP1, AQP2, and AQP9 in the testicular tissue of *Leptodactylus podicipinus*. (**A**,**C**,**E**): reproductive period; (**B**,**D**,**F**): non-reproductive period. AQP1 staining can be seen in germinative cells, Leydig, Sertoli, blood vessels (**A**), and in the intratesticular ducts (**B**). AQP2 labels germinative cells, Leydig cells (**C**), and intratesticular ducts (**D**). AQP9 immunoreaction can be seen only in intratesticular ducts of the reproductive period (**E**). Arrowheads = Sertoli cells; arrows = Leydig cells; circle = blood vessels; stars = intratesticular ducts. Magnification: 20× bar = 50 µm; 100× bar = 10 μm.

**Figure A3:**
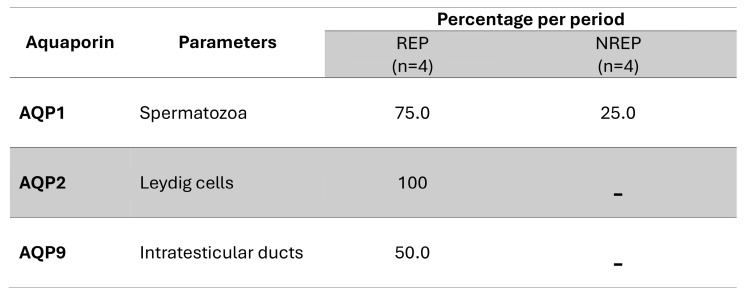
Percentage of testicular parameters with distinct aquaporin label per group of *L. podicipinus*. The percentage indicates the presence of the parameter in the individuals of each group (n = 4 per group); “-” indicates parameter absence.

The cellular localization of the three studied aquaporins was determined by immunohistochemistry. AQP1 localization was detected throughout the entire testicular tissue. The reaction was observed in all germline cells, even in the tails of bundled or free spermatozoa in the locular lumen. AQP1 was also detected in the Leydig and Sertoli cells (Figure 1A,B). Immunohistochemical images from both groups were compared qualitatively. AQP1 was labeled in spermatozoa in most images from the reproductive group (Figure 1A), whereas labeling was mostly absent in the non-reproductive group (Figure 1B and Figure A3). AQP1 was also observed in testicular blood vessels, with no apparent differences in labeling between the groups (Figure 1A,B).

Similar to AQP1 labeling, AQP2 exhibited immunoreactivity in the tail of spermatozoa, as well as in Sertoli cells. Leydig cell immunoreactivity for AQP2 was observed only in animals from the reproductive group (Figure 1C), whereas in the non-reproductive group, Leydig cells labeling was not detected in most of the images (Figure 1D and Figure A3). Additionally, AQP2 was detected in the epithelial cells of the intratesticular ducts of both groups (Figure 1C,D).

Immunoreactivity of AQP9 was predominantly observed in the epithelial cells of intratesticular ducts in animals from the reproductive group, with no distinct labeling evident in other regions of testicular tissue (Figure 1E,F and Figure A3).

**Figure 2:**
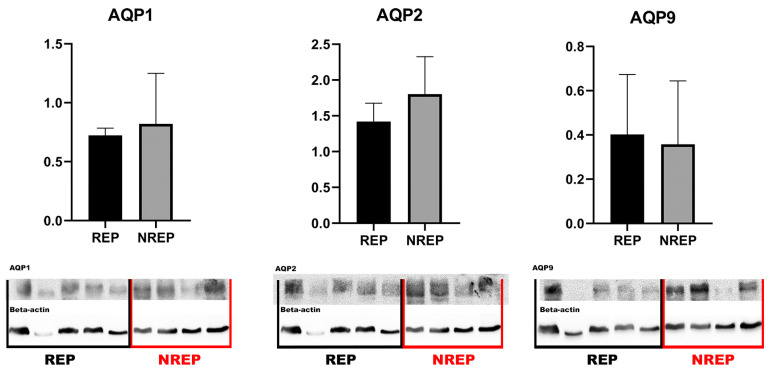
Western blot analysis of AQP1, AQP2, and AQP9 in *Leptodactylus podicipinus* testicle. Values are expressed as mean and standard deviation. REP (reproductive period), NREP (non-reproductive period).

Western blotting analysis for AQP1, AQP2, and AQP9 of the testicle extracts showed bands at 25–29 kDa in the majority of the samples analyzed (Figure 2). This finding corroborates the presence of these aquaporin within the testicular tissue of *L. podicipinus*, as further evidenced by the results obtained from the immunohistochemistry analysis. Notably, despite a thorough examination, there were no statistically significant differences in the mean levels of the aquaporins between the two periods studied.

## 4. Discussion

In this study, we not only elucidated the immunolocalization of AQP1, AQP2, and AQP9 in anuran testicular tissue but also incorporated Western blotting analysis, thus providing a multifaceted approach to characterizing aquaporin expression in this context. It is noteworthy that, while the aquaporin family has been investigated across various zoological groups, including fish, amphibians, birds, and mammals [31,41,42,43,44], our study makes a distinct contribution by integrating immunolocalization and Western blotting techniques to examine aquaporin in amphibian testes.

In the anuran species *Dryophytes chrysoscelis*, AQP1 was detected in the testis, expressed in testicular tissue and Leydig cells but not in germ cells and ejaculated spermatozoa, indicating that AQP1 is related to support cells and testicular development but not closely associated with mature spermatozoa [17]. In *L. podicipinus*, the findings contrast those for *D. chrysoscelis* because AQP1 is closely linked to both germinative and testicular tissue, indicating its presence in both support and spermatogenic cells. The difference seen could be a result of the interspecific variability, as this is common in anuran reproduction with a wide range of modes and strategies. Also, the two species could be differently influenced by factors such as climate, as temperature parameters are responsible for changes in aquaporin dynamics in *D. chrysoscelis*, a temperate species, encountered in North America (Canada and U.S), whereas *L. podicipinus*, a tropical species, predominantly inhabits South America (Brazil, Argentina, Bolivia, Paraguay, and Uruguay), and environmental factors such as temperature, rainfall, and photoperiod are commonly related to changes in frog reproduction [11,12].

In the fish species *Sparus aurata*, AQP1 appears to be specifically associated with early germ cells like spermatids and spermatozoa. The association suggests a potential role in addressing the metabolic requirements during the advanced stage of the spermatogenic process [44]. This study also identified AQP1 as being associated with germinative cells and frequently observed in the tails of spermatozoa. These observations support the hypothesis that AQP1 plays a pivotal role in the metabolic processes of spermatogenesis, particularly in spermiogenesis. Additionally, the strong labeling of AQP1 in spermatozoa from the reproductive group indicates its potential involvement in sperm maturation and motility.

Aquaporins exhibit varied characteristics denoting conservation and variation across the zoological groups; in goose testes, AQP1 is expressed in testicular blood vessels but not in Leydig cells [43]. In the tested anuran species, AQP1 was present in both the testicular blood vessels and Leydig cells, underscoring the conservation of AQP1’s role in blood regulation. However, there is variation in its expression in anuran Leydig cells, such which was not detected in birds.

Regarding AQP2, this aquaporin has been described in the turkey reproductive ducts and is considered to be involved in the maturation of avian spermatozoa [45]. Similar to birds, *L. podicipinus* AQP2 is present in spermatozoa; however, AQP1 seems to play a more significant role in the maturation process. This is evidenced by the strong labeling observed in both early and late germ cells, as well as in supporting cells such as Sertoli, along with the absence of labeling in sperm from the non-reproductive periods. These findings suggest that AQP1 in anurans may serve as a marker for the final stage of sperm maturation.

AQP2 was exclusively found in Leydig cells in animals from the reproductive group. This result may be attributed to the seasonal variation of vasotocin, a peptide that regulates AQP2 expression in anurans [39,41,46]. In addition to its involvement in various reproductive activities, such as vocalization, amplexus, and male agonistic behavior, vasotocin is predominantly expressed during the reproductive period of anurans, varying with steroid sex hormone levels that are regulated by somatic cells such as Leydig cells [46,47]. The observation of AQP2 labeling in Leydig cells only during the reproductive period suggest that vasotocin, which has been previously shown to regulate AQP2 in anurans [39,41,46], may play a role in this regulation within the reproductive tract. Although vasotocin was not directly tested in this study, these findings point to a potential connection between AQP2 and seasonal behaviors throughout the reproductive cycle of *L. podicipinus*.

AQP9 was detected in the interstitial tissues of *S. aurata* testis, specifically in Leydig cells [44]. However, the function of AQP9 regarding the Leydig cells remains unknown. In contrast, in *L. podicipinus* testis, AQP9 was found only on the intratesticular ducts, with no presence observed in interstitial tissue or germ cells. According to the results, AQP9 may be more closely associated with the transport of spermatozoa rather than participating in their maturation process. Specifically, the majority of AQP9 labeling in *L. podicipinus* testis was found in animals from the reproductive period, indicating that the function and localization of AQP9 may be closely related to the transportation and regulation of mature spermatozoa, as seen in the reproductive tract of mammals, where it is found in the epididymis of rodents and carnivores [19,42].

The localization of proteins is crucial for their function, especially in dynamically regulated processes such as reproduction. Our immunohistochemical observations indicate that aquaporin localization changes during the reproductive cycle. This suggests that aquaporins play specific and contextual roles in reproductive physiology, with high expression in critical locations during reproductive periods. The differential localization of these proteins can offer valuable insights into the molecular mechanisms regulating reproduction in anuran species, thereby aiding hypothesis formulation and guiding future research in this area.

The Western blot results for the studied aquaporin showed bands at 29–30 kDa, which align closely with existing results in the literature. In *D. chrysoscelis*, AQP1-type aquaporin was reported at 26 kDa [17], slightly lower than the results observed in *L. podicipinus*. For *Hyla japonica*, an AQP2-type aquaporin was described in urinary bladder tissue with bands at 30–31 kDa, similar to findings in *Rana catesbeiana*, *R. nigromaculata*, and *Bufo japonicus* [39,48]. The observed variations in the molecular weights of aquaporins may arise from differences in amino acid sequences, post-translational modifications, or tissue-specific isoforms. Additionally, limited data are available to describe the characteristics of AQP9 in anurans. Although the molecular weights of aquaporins provide valuable insights into their structural features, comprehensive studies encompassing functional assays, protein interactions, and expression patterns are necessary to unravel the complexities of AQP biology across different species and tissues.

Regarding Western blotting results, no statistically significant differences were observed between the reproductive and non-reproductive seasons, indicating that the differences observed were related to the location of the AQPs and not their quantity, as confirmed by the IHC analysis. Western blotting is effective for measuring global protein levels in tissue samples, but it does not detect variations in protein localization that are more apparent with immunohistochemistry. Furthermore, aquaporin expression can be regulated post-translationally, affecting their localization and functionality without altering the total levels detectable by Western blotting.

The protein levels obtained from the blot analysis showed no different values of AQP1 and AQP2 in animals from the non-reproductive period, which aligns with the immunohistochemical labeling results of spermatozoa and Leydig cells, as there are no statistical differences. The immunohistochemical differences were specific to specific cells and not to entire tissues. Because the labeling was predominantly found in other cells, the quantification of these aquaporins between the stages of reproduction could be related to the water conservation function rather than their reproductive and hormonal function, as these two functions are not mutually exclusive.

The aquaporin family in anurans has been studied for a long time, starting with the hypothesis related to the cellular water channels [16]. The present molecular results contribute to the understanding of aquaporin diversity within the anuran group by providing the molecular weight of AQPs from *L. podicipinus* and supplementing the previously reported molecular weights of the aquaporins HC-1 and AQPh2 from *D. chrysoscelis* and *H. japonica* [40,48]. This diversity is also related to the range of environmental behavior within the group, as reflected in physiological mechanisms such as water balance via aquaporins influenced by environmental or hormonal factors [16]. This is evident in the thermally specific expression of *D. chrysoscelis* HC-1 and HC-3 in osmoregulation organs [16,40]. Conversely, in *L. podicipinus* testicles, there was no significant difference in the quantity of AQP expression between periods, despite tissue expression variation. Although the organs originate from different systems, the lack of variation in *L. podicipinus* may reflect the stable and tropical environment that the individuals inhabit.

Furthermore, extended research and testing are necessary to determine whether the difference is related only to the localization of the aquaporins or if there is indeed a differential expression of these proteins throughout the reproductive cycle of the species.

## 5. Conclusions

This study is the first to describe the expression of AQP1, AQP2, and AQP9 in the testis of an anuran species. These AQPs were expressed in spermatozoa, Leydig cells, and intratesticular ducts mainly during the reproductive season. These observations underscore the impact of reproductive status on aquaporin expression. In conclusion, while this study advances our understanding of reproductive dynamics in anurans, it also underscores the need for further research to fully elucidate the cellular and physiological roles of aquaporin in this context.

## Figures and Tables

**Figure A2:**
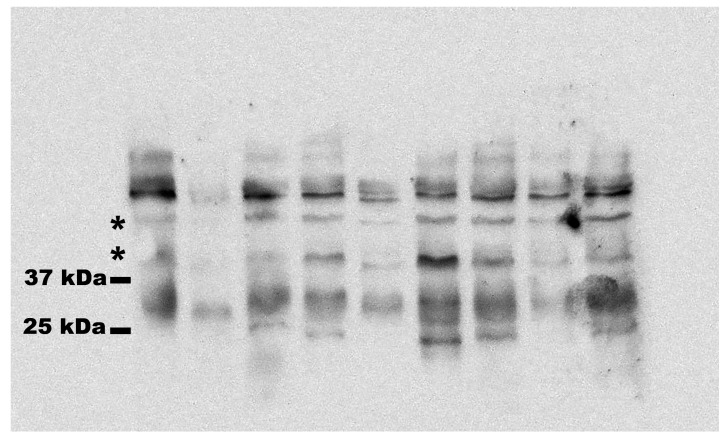
Full-range Western blot membrane of AQP1 from testis tissue of *L. podicipinus*. “*” unspecific bands.

## Data Availability

The Data is contained within the article.
